# Site-Specific Management of Miscanthus Genotypes for Combustion and Anaerobic Digestion: A Comparison of Energy Yields

**DOI:** 10.3389/fpls.2017.00347

**Published:** 2017-03-17

**Authors:** Andreas Kiesel, Christopher Nunn, Yasir Iqbal, Tim Van der Weijde, Moritz Wagner, Mensure Özgüven, Ivan Tarakanov, Olena Kalinina, Luisa M. Trindade, John Clifton-Brown, Iris Lewandowski

**Affiliations:** ^1^Department Biobased Products and Energy Crops, Institute of Crop Science, University of HohenheimStuttgart, Germany; ^2^Institute of Biological, Environmental and Rural Sciences, Aberystwyth UniversityAberystwyth, UK; ^3^Department of Plant Breeding, Wageningen UniversityWageningen, Netherlands; ^4^Faculty of Agriculture and Natural Sciences, Konya Food and Agriculture UniversityKonya, Turkey; ^5^Russian State Agrarian University–Moscow Timiryazev Agricultural AcademyMoscow, Russia

**Keywords:** biogas, harvest time, biomass, yield, energy yield, substrate-specific methane yield, moisture content

## Abstract

In Europe, the perennial C_4_ grass miscanthus is currently mainly cultivated for energy generation via combustion. In recent years, anaerobic digestion has been identified as a promising alternative utilization pathway. Anaerobic digestion produces a higher-value intermediate (biogas), which can be upgraded to biomethane, stored in the existing natural gas infrastructure and further utilized as a transport fuel or in combined heat and power plants. However, the upgrading of the solid biomass into gaseous fuel leads to conversion-related energy losses, the level of which depends on the cultivation parameters genotype, location, and harvest date. Thus, site-specific crop management needs to be adapted to the intended utilization pathway. The objectives of this paper are to quantify (i) the impact of genotype, location and harvest date on energy yields of anaerobic digestion and combustion and (ii) the conversion losses of upgrading solid biomass into biogas. For this purpose, five miscanthus genotypes (OPM 3, 6, 9, 11, 14), three cultivation locations (Adana, Moscow, Stuttgart), and up to six harvest dates (August–March) were assessed. Anaerobic digestion yielded, on average, 35% less energy than combustion. Genotype, location, and harvest date all had significant impacts on the energy yield. For both, this is determined by dry matter yield and ash content and additionally by substrate-specific methane yield for anaerobic digestion and moisture content for combustion. Averaged over all locations and genotypes, an early harvest in August led to 25% and a late harvest to 45% conversion losses. However, each utilization option has its own optimal harvest date, determined by biomass yield, biomass quality, and cutting tolerance. By applying an autumn green harvest for anaerobic digestion and a delayed harvest for combustion, the conversion-related energy loss was reduced to an average of 18%. This clearly shows that the delayed harvest required to maintain biomass quality for combustion is accompanied by high energy losses through yield reduction over winter. The pre-winter harvest applied in the biogas utilization pathway avoids these yield losses and largely compensates for the conversion-related energy losses of anaerobic digestion.

## Introduction

Miscanthus is a resource-use efficient, high-yielding perennial C4 grass species native to East Asia, including China, Korea, Taiwan, and Japan (Lewandowski and Schmidt, 2006; Clifton-Brown et al., 2015). The cultivation of miscanthus is characterized by its perennial nature and low nitrogen-fertilization demand, due to its effective nutrient recycling system (Christian et al., 2008; Strullu et al., 2011; Cadoux et al., 2012). This leads to a generally benign environmental profile, often associated with soil carbon sequestration (McCalmont et al., 2017). For these reasons, miscanthus biomass utilization generally shows a low global-warming and resource-depletion potential (Felten et al., 2013; Styles et al., 2015; Meyer et al., 2016). Despite these positive aspects, the miscanthus cultivation area is still rather small in Europe, mainly due to its high establishment costs and the current lack of valorisation options.

The only cultivar presently commercially available is *Miscanthus x giganteus* (Mxg), a natural, sterile hybrid of *Miscanthus sacchariflorus* and *Miscanthus sinensis*, which was introduced into Europe in 1935 (Greef et al., 1997; Clifton-Brown et al., 2015). As Mxg is sterile, only clonal propagation is possible. This is costly and does not allow for crop development by conventional breeding. Therefore, miscanthus breeding for European conditions is mainly focussing on the groups *M. sinensis, M. sacchariflorus*, and *Miscanthus floridulus*, which offer broad genetic variability and the possibility of reducing establishment costs through economical, seed-based propagation (van der Weijde et al., 2013; Clifton-Brown et al., 2017). In the EU project OPTIMISC (FP7 No. 289159), early stage crossings from the ongoing miscanthus breeding programmes of Aberystwyth (IBERS) and Wageningen University (WUR) were tested at several locations, under different stress conditions and for various utilization options (Lewandowski et al., 2016).

Combustion is one of the most common utilization options for miscanthus biomass, but production of cellulosic ethanol and anaerobic digestion were identified as promising alternatives (van der Weijde et al., 2013, 2017b; Mayer et al., 2014; Wahid et al., 2015); Kiesel and Lewandowski, 2017. For each utilization option, ideal harvest time is of crucial importance to maintain high quality and yield. For combustion, the harvest time is delayed to reduce the contents of moisture, ash, and critical elements (Iqbal and Lewandowski, 2014). However, there is a trade-off here between yield and quality, as leaf losses occur over winter and lead to a decrease in biomass yield (Iqbal et al., under review). For biogas, an early green harvest delivers a higher quality, since the substrate-specific methane yield decreases with ongoing lignification (Kiesel and Lewandowski, 2017). Here again there is a trade-off, as a very early green harvest delivers a lower yield, due to insufficient utilization of the vegetation period, and also impairs the crop growth the next season due to insufficient relocation of carbohydrates (Purdy et al., 2015; Kiesel and Lewandowski, 2017). The latter is referred to as “cutting tolerance,” which has been defined for miscanthus as the ability of the crop to recover from an early green harvest without yield reductions in the following year (Kiesel and Lewandowski, 2017). As the ideal harvest time is a compromise between yield, quality, and cutting tolerance in both utilization options, the development of the energy yield (which includes biomass yield and quality) needs to be quantified throughout the year. In addition, a comparison of energy yield between combustion and anaerobic digestion is required to establish the loss associated with the generation of the higher-value product. In this case, biomethane—which is upgraded solid biomass—is seen as a higher-value product. As a gaseous fuel, it has a broader range of applications, including transport fuel, and its application in combined heat and power generation is easier, including transport, storage, and utilization of biomethane in existing natural gas infrastructure.

In addition to harvest time, the genotype also affects biomass quality. For combustion, genotypes with low contents of moisture, ash and critical elements at harvest are optimal, while for anaerobic digestion a low degree of lignification and ease of digestibility is preferred. Iqbal and Lewandowski (2014) found notable genotypic differences in contents of ash and critical elements, which can be partly attributed to genotypic differences in nutrient relocation and leaching of soluble elements. For biogas and ethanol utilization, van der Weijde et al. (2017b) observed both a higher saccharification potential and substrate-specific methane yield in less lignified genotypes. Location may also play a crucial role. For example, drought conditions can increase the saccharification potential of miscanthus biomass (van der Weijde et al., 2017a).

The objective of this paper is (i) to identify the effect of genotype, environment and harvest time on yield and biomass quality for anaerobic digestion and combustion and (ii) to compare the energy yield of both pathways throughout the year. For this purpose, five miscanthus genotypes from the OPTIMISC multi-location field trials were sampled at monthly intervals throughout the end of the vegetation period until final harvest in spring at the locations in Adana (Turkey), Moscow (Russia), and Stuttgart (Germany). Energy yield, biomass yield, and a number of quality parameters (including substrate-specific methane yield) were assessed and compared for each sampling date. This allows identification of site-specific optimization potentials for each utilization option. This paper focuses on biomass quality for anaerobic digestion, but also includes some basic quality criteria relevant for the energy yield via combustion, such as moisture and ash content. A detailed combustion quality analysis, including the content of critical elements, and a quantification of the trade-off between yield and biomass quality can be found in Iqbal et al. (under review). Further the net energy yield via anaerobic digestion and combustion, which considers moisture and ash content, was assessed and compared, to allow site-specific identification of the best suited harvest date for each utilization option.

## Materials and methods

### Field trial

The field trial was established in 2012 as part of the EU-financed project OPTIMISC (FP7 No. 289159) to compare 15 miscanthus genotypes at 6 sites across Europe and Russia: at Aberystwyth (UK), Adana (Turkey), Moscow (Russia), Potash (Ukraine), Stuttgart (Germany), and Wageningen (Netherlands). It was set up in a randomized block design with three biological replications at each location. A detailed description of the field trial including genotypes used, soil and climatic conditions can be found in Kalinina et al. (under review) and Lewandowski et al. (2016). For this paper, five genotypes (best yields) and three locations (contrasting climates) were selected, where at least one representative from each miscanthus group (species) was included. The selected genotypes are shown in Table 1 and the chosen locations were Adana, Moscow, and Stuttgart.

**Table 1:** **Miscanthus “genotypes” used in this investigation (Lewandowski et al., **2016**)**.

**Genotype ID**	**Provider**	**Species**
OPM 3	IBERS	*Miscanthus sacchariflorus*
OPM 6	IBERS	*Miscanthus sinensis* x *Miscanthus sacchariflorus* hybrid
OPM 9	IBERS	*Miscanthus* x *giganteus*
OPM 11	IBERS	*Miscanthus sinensis* “Goliath”
OPM 14^*^	WUR	*Miscanthus sinensis*

* strictly speaking, OPM 14 is a “within species” hybrid rather than a true genotype, but for convenience is referred to throughout as a “genotype.”

The genotypes were sampled at intervals of 1–2 months from the end of vegetation period until the final harvest in spring (Table 2). In Moscow and Stuttgart, the final harvest was performed in March. In Adana, it took place in January, because the plants had already started to regrow. In Moscow, sampling was interrupted after September to the final harvest, because the aboveground parts of the crop were completely killed by a harsh frost a few days before the sampling date in September.

**Table 2:** **Sampling dates and location characteristics. na = not applicable/no sampling performed**.

**Location**	**Latitude Longitude Altitude (m)**	**Sampling date**					
		**1 August (A)**	**2 September (S)**	**3 October (O)**	**4 November (N)**	**5 January (J)**	**6 March (M)**
	37.00						
Adana	35.00	20.8.14	20.9.14	20.10.14	20.11.14	20.01.15	na
	27						
	55.50						
Moscow	37.33	20.8.14	20.9.14	na	na	na	13.03.15
	140						
	48.74						
Stuttgart	8.93	28.8.14	25.9.14	23.10.14	27.11.14	22.01.15	18.03.15
	463						

**Figure 1:**
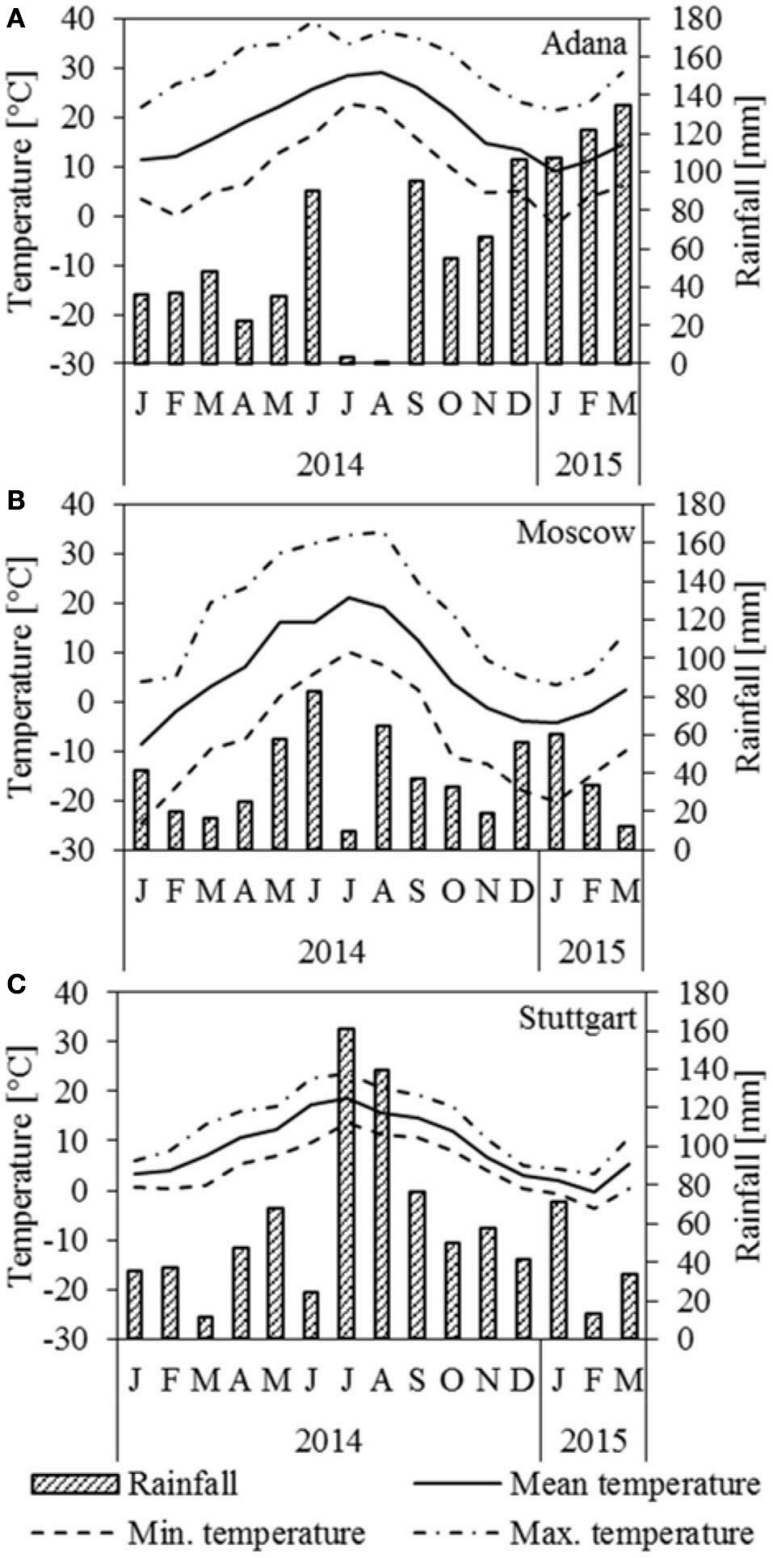
**Temperature and rainfall at the location (A)** Adana, **(B)** Moscow, and **(C)** Stuttgart for 2014 and the first 3 months of 2015.

Figure 1 depicts rainfall and temperature data for the three locations Adana, Moscow, and Stuttgart. In Adana, a seasonal drought period occurred in July and August. There was only little frost in January 2015 (Figure 1A). In Moscow, July was particularly dry and the plants faced a serious drought (Figure 1B). The winter started very abruptly at the end of September with harsh frosts and the crop was frozen most of the time until March. In Stuttgart, June was abnormally dry, but in the following 2 months the rainfall was higher than usual (Figure 1C). Overall, the winter 2014/2015 was mild, but there was a frost period in January and February 2015.

### Biomass yield estimation

On each sampling date, eight tillers were collected randomly from each genotype. The samples were taken from the second outer row to avoid damaging the core plot, which was used for final harvest biomass yield estimation. To ensure the samples were taken randomly, a bar with marks every 60 cm was used. The tiller closest to each 60-cm mark was collected. The central four m^2^ of each plot were used for biomass yield estimation at final harvest in January (Adana) or March (Moscow, Stuttgart) and harvested manually using a hedge trimmer or sickle bar mower. Before the final harvest, another eight tillers were collected randomly. All samples were dried to constant weight at 60°C in a cabinet dryer and fresh and dry weight was recorded. Dry matter content and reciprocal value moisture content were calculated according to weight loss. Based on the weight of the eight tillers at each sampling date and the biomass yield at final harvest, the dry and fresh matter yield at each sampling date was calculated (Equation 1). The dry matter yield at each sampling date was calculated using a ratio of the stem weights at the sampling date and the final harvest. The details of this calculation are described by Nunn et al. (under review).

(1) $$\begin{array}{l} Yield_{n}=\;\frac{Weight\;8\;tillers_{n}}{Weight\;8\;tillers_{m}}\;*\;Yield_{m} \end{array}$$

where

Yield_*n*_ = Biomass yield at sampling date n

Weight 8 tillers_n_ = Weight of eight tillers at sampling date n

Weight 8 tillers_m_ = Weight of eight tillers at final harvest in March (January at Adana)

Yield_*m*_ = Biomass yield at final harvest in March (January at Adana), estimated at central 4 m^2^.

### Laboratory analysis

All dried samples were send to University of Hohenheim, where all further analysis have been performed. The biomass samples were milled in a cutting mill SM 200 (Retsch, Haan) using a 1 mm sieve before further laboratory analysis. The ash content of all samples was assessed by incineration in a muffle kiln at 550°C for 4 h according to VDLUFA book III method 8.1 (Naumann and Bassler, 1976/2012).

Content of neutral detergent fiber (NDF), acid detergent fiber (ADF) and acid detergent lignin (ADL) was estimated by near infrared spectroscopy (NIRS). Calibration and validation samples were analyzed using an ANKOM^2000^ Fiber Analyzer and Daisy II Incubator (ANKOM Technology, Macedon, USA) according to VDLUFA book III method 6.5.1 (NDF), 6.5.2 (ADF), and 6.5.3 (ADL) (Naumann and Bassler, 1976/2012). The standard error of the NIRS calibration (SEC) and prediction (SEP) and the *R*^2^ of the NIRS calibration and validation are shown in Table 3. The ADL content is considered lignin. Cellulose content was calculated by subtracting ADL from ADF, and hemicellulose by subtracting ADF from NDF.

**Table 3:** **NIRS calibration and validation statistics**.

	**Calibration**			**Validation**		
	**Number of samples**	**Standard error of calibration**	***R*^2^**	**Number of samples**	**Standard error of prediction**	***R*^2^**
NDF	160	1.2672	0.953	20	2.345	0.858
ADF	160	1.3331	0.959	20	2.699	0.834
ADL	160	0.6492	0.888	20	0.773	0.706

The specific methane yield (SMY) was measured in a biogas batch test at 39°C according to VDI guideline 4630. The biogas batch method was certified by the KTBL and VDLUFA inter-laboratory comparison test in 2014 and 2015 and is described in detail in Kiesel and Lewandowski (2017). The SMY was analyzed by using 200 mg oDM of the dried and milled biomass samples and 30 g of inoculum, which contained various macro- and micronutrients according to Angelidaki et al. (2009). The fermentation was performed for 35 days in gastight fermentation flasks and the biogas production was measured by the pressure increase using a HND-P pressure meter (Kobold Messring GmbH, Hofheim). The methane content of the biogas was measured by using a GC 2014 gas chromatograph (Shimadzu, Kyoto). However, for capacity reasons it was not possible to analyse all samples. Therefore, a minimum of one field replication of each genotype from each sampling date and each location was selected randomly to be analyzed. All samples were analyzed in one run of the biogas batch test to assure statistical soundness. A randomized block design with four technical replicates was applied. For capacity reasons, the batch test had to be split into two water baths. Replicates 1 and 2 were analyzed in one and replicates 3 and 4 in the other.

The methane yield per hectare was calculated based on estimated dry matter yield (DMY), ash content and SMY. As the SMY was mostly analyzed for only one of the three field replications, this value (or the average of all field replications analyzed) was assumed for all three field replications.

The net energy yield of anaerobic digestion was calculated by multiplying the methane yield per hectare by the calorific value of methane (35.883 MJ m^−3^) as shown in Equation (2). The net energy yield of combustion was calculated according to Equation (3), in which an average calorific value of 18 MJ kg^−1^ for dry miscanthus biomass (Kołodziej et al., 2016) and 2.443 MJ kg^−1^ enthalpy of water vaporization was assumed. The net energy yield is considering not only ash and moisture content of the biomass, but also the energy required to evaporate the incorporated water.

(2) $$\begin{array}{r} Net\;Energy\;Yield_{Anaerobic\;digestion}=CV_{Methane}\;*\;SMY\\ {}*\;DMY\;*\left(1-AC\right) \end{array}$$

(3) $$\begin{array}{r} Net\;Energy\;Yield_{Combustion}=CV_{Miscanthus}*DMY*\left(1-AC\right)\\ -\;EE_{Water}*FMY*MC \end{array}$$

where

CV_Methane_ = calorific value of methane (35.883 MJ m^−3^)

SMY = substrate-specific methane yield

DMY = dry matter yield of miscanthus

AC = ash content of the miscanthus biomass

CV_Miscanthus_ = calorific value of dry miscanthus biomass (18 MJ kg^−1^)

EE_Water_ = evaporation enthalpy water (2.443 MJ kg^−1^)

FMY = fresh matter yield of miscanthus

MC = moisture content of the miscanthus biomass.

### Statistical analysis

Statistical analysis was performed using the software SAS version 9.4 (SAS Institute Inc., Cary, North Carolina). The program “Procmixed” was used and a mixed model applied (Equation 4). A test on homogeneity of variance and normal probability of residues was performed. The effects were tested at a level of probability of α = 0.05.

(4) $$\begin{array}{r} y=\mu +\text{ Loc }+\text{ Geno}+\text{Loc}*\text{Geno}+\text{HD}\left(\text{Loc}\right)\\ +\text{ Geno}*\text{HD}\left(\text{Loc}\right)\;+\text{ e} \end{array}$$

where

μ = general mean effect

Loc = effect of location (Adana, Moscow, Stuttgart)

Geno = effect of genotype (OPM 3, 6, 9, 11, 14)

Loc*Geno = effect of interaction of location and genotype

HD(Loc) = effect of location specific sampling date

Geno * HD(Loc) = effect of interaction of genotype and location specific sampling date

e = residual error.

## Results

In the following chapter, the results of each genotype at each harvest date and location are shown in figures, but for clarity reasons letters are displayed only for the sampling dates per location [HD(Loc)]. Tables with means for genotype and location at each harvest date and the respective letter displays are given in the supplementary material.

### Fixed effects

Location (Loc) and sampling date per location [HD(Loc)] showed highly significant impacts on all traits analyzed (Table 4). Genotype (Geno) and interaction of location and genotype (Loc*Geno) had a highly significant impact on quality parameters and a still significant impact on yield-related parameters, such as methane yield per hectare and net energy yield of biogas and combustion (Table 4). This may be influenced by the high variance in yield, caused by the fairly rough yield estimation using eight tillers. The interaction of genotype and sampling date per location [Geno*HD(Loc)] showed a significant impact only on dry matter, hemicellulose and lignin content. Again, the variance due to the small sampling size of eight tillers may have been too high. However, larger sampling size was not feasible to avoid impact on the field trial.

**Table 4:** *****P***-values of fixed effects**.

	**Yield**	**Dry matter content**	**Ash content**	**Cellulose content**	**Hemicellulose content**	**Lignin content**	**SMY**	**Methane yield per hectare**	**Net energy yield biogas**	**Net energy yield combustion**
Loc	<0.001	<0.001	<0.001	<0.001	<0.001	<0.001	<0.001	<0.001	<0.001	<0.001
Geno	0.010	<0.001	<0.001	<0.001	<0.001	<0.001	<0.001	0.037	0.039	0.006
Loc^*^Geno	0.006	0.001	<0.001	<0.001	<0.001	<0.001	0.015	0.029	0.030	0.036
HD(Loc)	<0.001	<0.001	<0.001	<0.001	0.007	<0.001	<0.001	<0.001	<0.001	<0.001
Geno^*^ HD(Loc)	ns	<0.001	ns	ns	0.001	0.037	ns	ns	ns	ns

### Biomass yield and dry matter content

**Figure 2:**
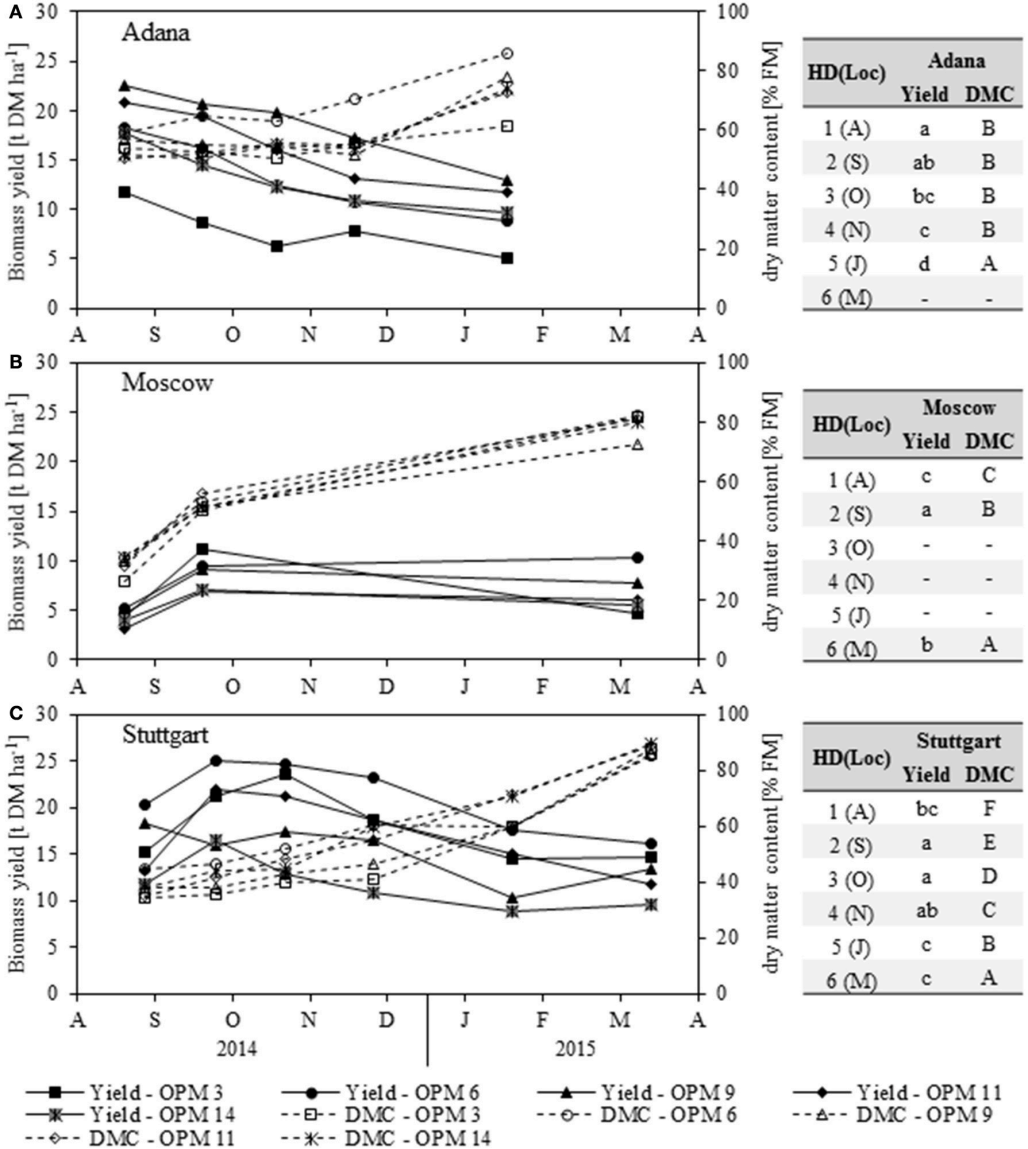
**Biomass dry matter yield (Yield) and dry matter content (DMC) of each genotype [OPM 3, 6, 9, 11, 14) for each sampling date (1 = August (A), 2 = September (S), 3 = October (O), 4 = November (N), 5 = January (J), 6 = March (M)] at the locations (A)** Adana, **(B)** Moscow and **(C)** Stuttgart. Tables include the letter display for the sampling date per location [HD(Loc)] for the traits yield and DMC. Different lower- (Yield) and upper-case (DMC) letters indicate significant differences at a probability level of α = 0.05 for sampling dates at a specific location.

There was a large difference in biomass yield development throughout the year between the Adana location (the warmest in this study) and the other two locations (Figure 2).

In Adana, the biomass yield was significantly highest in August and then declined steadily until final harvest in March (Figure 2A). The highest biomass yields at each sampling date were found for OPM 9, which declined from 22.6 t DM ha^−1^ in August to 13.0 t DM ha^−1^ in March. Significantly lower biomass yields were found in OPM 3. The biomass yields of all the other genotypes showed no significant differences.

In Moscow, significantly higher biomass yields were found in September (Figure 2B) and OPM 3 (11.2 t DM ha^−1^) was the highest-yielding genotype in this month (Figure 2B). At final harvest in March, OPM 6 and 9 had the highest DM yields (10.3 and 7.7 t DM ha^−1^). These had stayed quite stable over winter, while the yield of OPM 3 had declined severely to 4.7 t DM ha^−1^.

In Stuttgart, the biomass yield behavior was similar to that in Moscow. Significantly higher biomass yields were found in September and October and all genotypes showed significant yield losses over winter (Figure 2C). The highest DM yields were found for OPM 6, which increased to 25.0 t DM ha^−1^ in September and then decreased to 16.2 t DM ha^−1^ in March. However, the biomass yields of OPM 6 were only significantly different from OPM 14. Interestingly, OPM 9 (Mxg) showed comparatively low biomass yields in the course of the year but an increase from January to March (10.2–13.4 t DM ha^−1^). Yield measurement in OPM 9 was difficult due to the shape of the crop (center of the plot was considerably higher than the border rows), which may have led to an underestimation of yield, especially in January. However, the final harvest in March was performed at the center of the plot and therefore delivered reasonable biomass yields.

The dry matter content (DMC) increased steadily at all locations throughout the year and the significantly highest DMC was recorded at final harvest in March/January (Figure 2). In Adana, OPM 6 showed the highest DMC throughout the year and at final harvest in January (Figure 2A). It was also the only genotype in Adana that achieved a DMC of above 80% FM at final harvest, which is crucial for safe storage of the biomass. In Moscow, no significant differences in DMC were detected between the genotypes, but OPM 9 was the only genotype with a DMC of below 80% FM at final harvest (Figure 2B). In Stuttgart, OPM 6 showed the highest DMC from August to November, but further drying was hindered by lodging of the crop (Figure 2C). In January, OPM 11 and 14 showed the highest DMC. However, the differences in DMC at final harvest in March were very small, due to good weather conditions (frost in winter, dry before harvest).

### Methane yield and SMY

**Figure 3:**
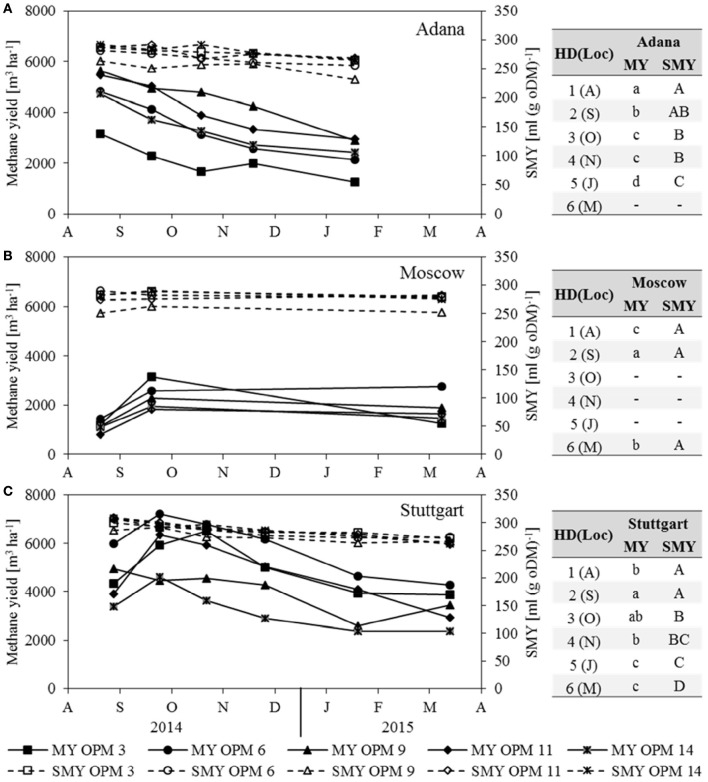
**Methane yield (MY) and substrate-specific methane yield (SMY) for each genotype [OPM 3, 6, 9, 11, 14) and sampling date (1 = August (A), 2 = September (S), 3 = October (O), 4 = November (N), 5 = January (J), 6 = March (M)] at the locations (A)** Adana, **(B)** Moscow and **(C)** Stuttgart. Tables include the letter display for the sampling date per location [HD(Loc)] for the traits methane yield (MY) and substrate-specific methane yield (SMY). Different lower- (MY) and upper-case (SMY) letters indicate significant differences at a probability level of α = 0.05 for sampling dates at a specific location.

In Moscow, the substrate-specific methane yield (SMY) did not change significantly throughout the year (Figure 3B). In Adana and Stuttgart, it decreased significantly from August to final harvest in March (Figures 3A,C). However, the impact of the SMY on methane yield was only slight compared to that of biomass yield. It can be clearly seen that MY follows the same trend as dry matter yield and is therefore not described separately here.

The SMY of OPM 9 was the significantly lowest of all assessed genotypes at all locations. That of OPM 14 was very similar at all three locations, while that of OPM 9 and 11 was significantly higher in Stuttgart than in Adana and Moscow. The SMY of OPM 3 and OPM 6 was significantly lower in Adana than in Stuttgart, but there was no significant difference between Stuttgart and Moscow.

### Fibre and ash contents

**Figure 4:**
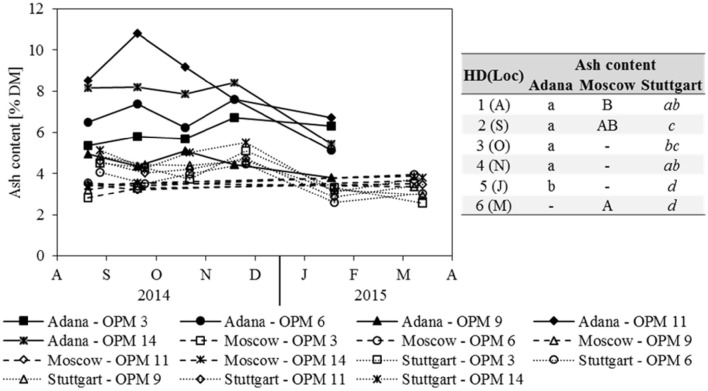
**Ash content for each genotype (OPM 3, 6, 9, 11, 14) and sampling date [1 = August (A), 2 = September (S), 3 = October (O), 4 = November (N), 5 = January (J), 6 = March (M)] at the three locations Adana, Moscow, and Stuttgart**. Tables include the letter display for the sampling date per location [HD(Loc)] for the traits ash content. Different lower- (Adana) and upper-case (Moscow) and italic (Stuttgart) letters indicate significant differences at a probability level of α = 0.05 for sampling dates at a specific location.

Ash content was strongly influenced by location and Adana showed the significantly highest ash contents at each sampling date (Figure 4). In Adana, the ash content only decreased significantly from November to January. In Stuttgart, a significant decrease was also observed from November to January and the biomass sampled in January and March had the significantly lowest ash content. In contrast, the ash content in Moscow increased slightly, but significantly, from August to March. Genotype OPM 11 showed the significantly highest ash content at Adana and OPM 14 at Stuttgart. In Moscow, no significant genotypic differences were recorded.

**Figure 5:**
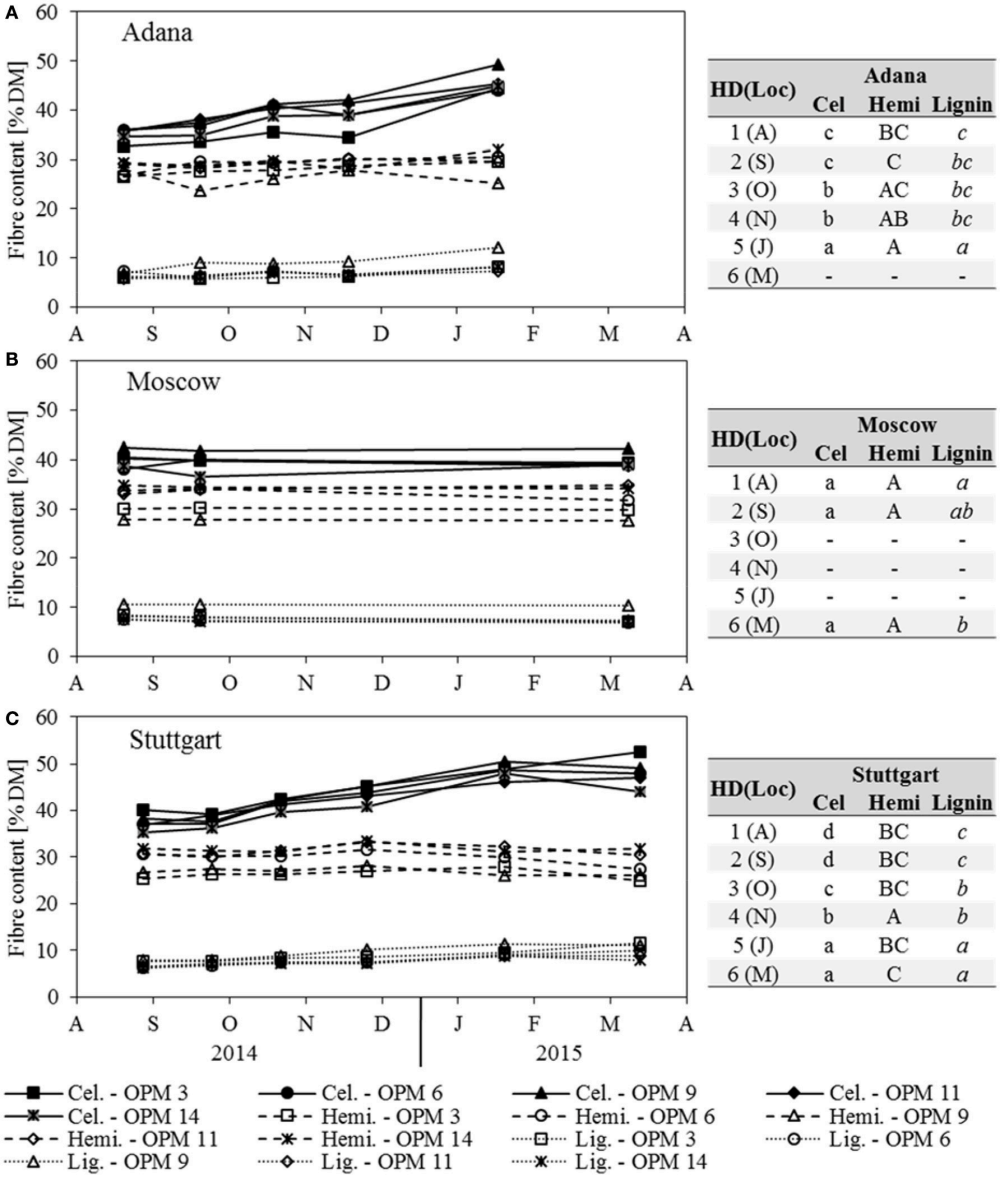
**Cellulose (Cel), hemicellulose (Hemi) and lignin content of each genotype (OPM 3, 6, 9, 11, 14) and sampling date [1 = August (A), 2 = September (S), 3 = October (O), 4 = November (N), 5 = January (J), 6 = March (M)] at the three locations (A)** Adana, **(B)** Moscow, and **(C)** Stuttgart. Tables include the letter display for the sampling date per location [HD(Loc)] for the traits cellulose, hemicellulose and lignin content. Different lower- (Cel) and upper-case (Hemi) and italic (Lignin) letters indicate significant differences at a probability level of α = 0.05 for sampling dates at a specific location.

The cellulose content increased steadily at Adana and Stuttgart, where the significantly highest contents were recorded for sampling dates January and March (Figure 5). All genotypes showed the significantly highest cellulose contents at Stuttgart, but those at Adana and Moscow were mostly not significantly different. Here, OPM 9 showed the significantly highest cellulose content of all genotypes (not significantly higher than OPM 11 in Adana). In Stuttgart, the significantly highest cellulose contents were found with OPM 6 and OPM 9. In Moscow, both cellulose and hemicellulose contents did not significantly change over the year; only a slight, but significant decrease in lignin was recorded.

In Adana, the hemicellulose content increased slightly with later sampling dates and the significantly highest hemicellulose content was found in January, but it was not significantly different from November and October (Figure 5A). In Stuttgart, the hemicellulose content increased slightly until November (significantly highest) and then decreased at the same rate (Figure 5C). At all locations, OPM 9 had the significantly lowest hemicellulose content, except OPM 3 at Stuttgart. The hemicellulose content of all genotypes was highest (mostly significantly) at the Moscow location.

The lignin content increased steadily with later sampling dates at the Adana and Stuttgart locations, where the significantly highest lignin contents were recorded in January and March (Figure 5). At all locations, OPM 9 showed the significantly highest lignin content, however it was not significantly higher than that of OPM 3 at Stuttgart.

### Net energy yields

**Figure 6:**
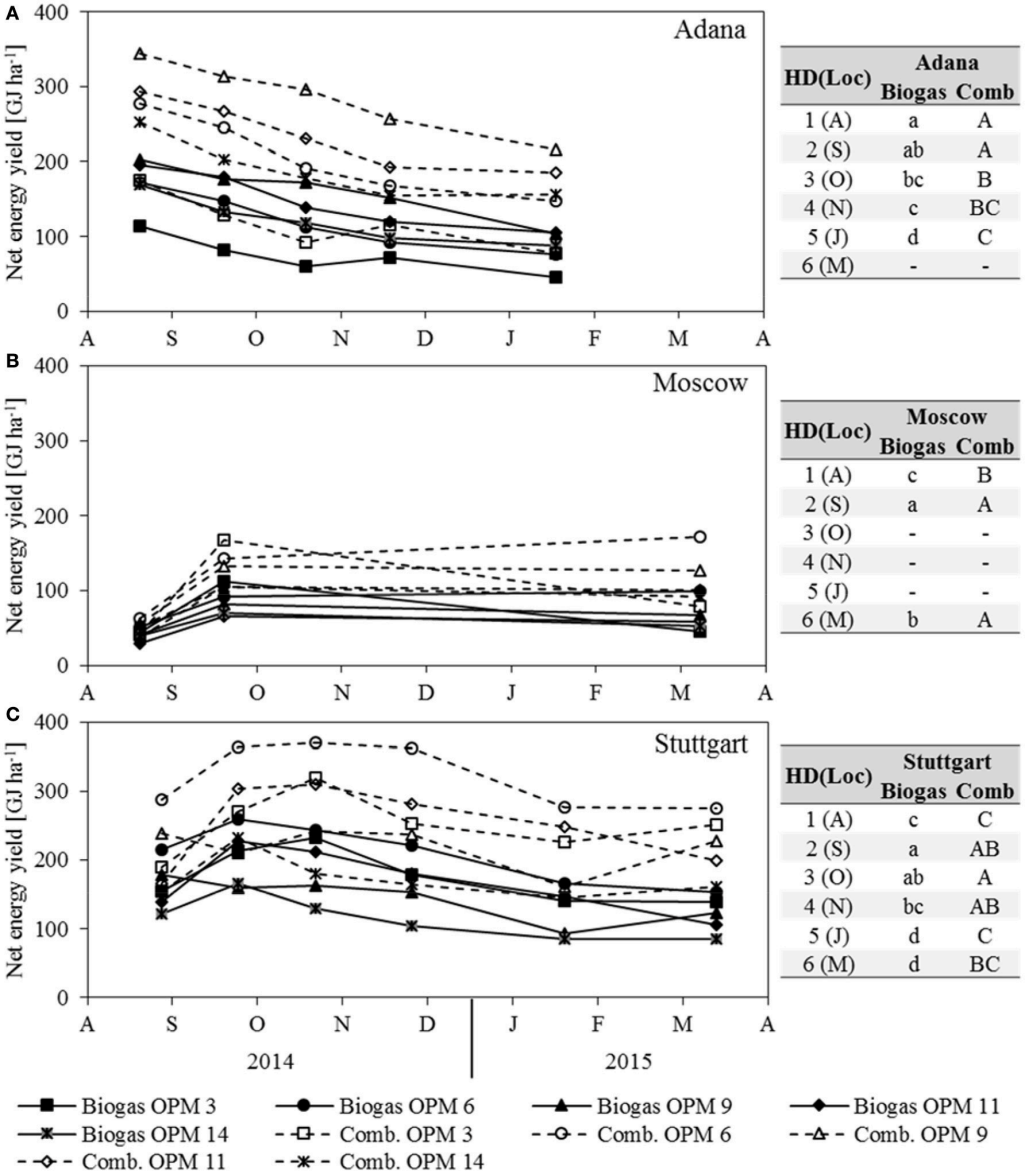
**Net energy yield of anaerobic digestion (Biogas) and combustion (Comb) of each genotype (OPM 3, 6, 9, 11, 14) and each sampling date [1 = August (A), 2 = September (S), 3 = October (O), 4 = November (N), 5 = January (J), 6 = March (M)] at the three locations (A)** Adana, **(B)** Moscow, and **(C)** Stuttgart. Tables include the letter display for the sampling date per location [HD(Loc)] for the net energy yield of anaerobic digestion (Biogas) and combustion (Comb). Different lower- (Biogas) and upper-case (Comb) letters indicate significant differences at a probability level of α = 0.05 for sampling dates at a specific location.

The net energy yield of anaerobic digestion is influenced by dry matter yield, SMY, and ash content, whereas the net energy yield of combustion is influenced by dry matter yield, moisture content and ash content. For both, dry matter yield has the largest impact. As the development of both net energy yields clearly follows that of dry matter yield, it is not described separately here (Figure 6). In Adana, the highest net energy yield of combustion and anaerobic digestion was recorded for OPM 9 in August at 344 and 203 GJ ha^−1^, respectively. At this location, the net energy yield of both combustion and anaerobic digestion decreased steadily, by 37 and 49% respectively, until final harvest in January. In Moscow, the genotypes with the highest net energy yield of combustion and anaerobic digestion in September were OPM 3 at 168 and 113 GJ ha^−1^ and OPM 6 at 143 and 92 GJ ha^−1^, respectively. While the net energy yield of OPM 3 decreased noticeably (−53% for combustion and −60% for anaerobic digestion), OPM 6 showed a net energy yield of combustion and anaerobic digestion of 172 and 99 GJ ha^−1^, respectively. In Stuttgart, the highest net energy yield of combustion was observed in October and of anaerobic digestion in September for OPM 6 at 370 and 259 GJ ha^−1^, respectively. Here, at final harvest in March, the energy yield of combustion and anaerobic digestion of OPM 6 was 275 and 154 GJ ha^−1^, respectively.

A comparison of the two energy yields shows that, on average over all locations, genotypes and sampling dates, anaerobic digestion delivers 65% of the energy yield of combustion. However, there are noteworthy differences between location, genotypes and harvest dates. Early sampling in August improves the net energy yield of anaerobic digestion through an increase in SMY, but impairs the net energy yield of combustion through a higher moisture content. In August, the average net energy yield of anaerobic digestion for all locations and genotypes was 75% that of combustion; in Stuttgart and Moscow even 79 and 83%, respectively. Late harvest in January or March leads to a decrease in SMY and improved quality for combustion (lower moisture content). At final harvest, the net energy yield of anaerobic digestion, averaged over all locations and genotypes, was 55% of that of combustion; for OPM 9 even as low as 52%.

## Discussion

The energy yields (used here synonymously with “net energy yield”) per hectare of combustion and anaerobic digestion are mainly influenced by the harvestable biomass yield per hectare, but are differentially sensitive to content of organic and inorganic compounds in the biomass. The different biomass fractions, e.g., moisture, ash, and lignin content, interact to produce a thermal calorific value (combustion) or substrate-specific methane yield (anaerobic digestion). In combustion, inorganics such as ash mainly reduce the combustible proportion of the yield, whereas vaporization of water consumes additional energy and reduces the calorific value. For this reason, moisture content has the strongest quality-related impact on the energy yield of combustion. Biomass quality for anaerobic digestion is mainly related to the organic composition, in particular the lignin content. Here the energy yield is directly measured by the substrate-specific methane yield (SMY) in a biogas batch test, which is therefore the sole determining quality factor. Other biomass quality characteristics, such as lignin content, are only used to explain differences in SMY. The moisture content is not relevant for the energy yield of anaerobic digestion, since it is already considered during estimation of dry matter yield. In both conversion pathways, ash content reduces the amount of combustible and digestible biomass to the same extent (SMY is also calculated on the basis of organic dry matter), therefore it is not discussed in the following section.

All these yield and quality traits are influenced by genotype, location, harvest date and interaction of genotype and location. The following sections first discuss the impacts of the above determinants on energy yields of combustion and anaerobic digestion and then the energy yields are compared.

### Factors influencing energy yield

In both utilization pathways, harvestable yield (standardized by calculating dry matter at the different harvest times) had the largest impact on energy yield. Since location, genotype, and harvest date all have an influence on harvestable dry matter yield, these also had a considerable impact on energy yield. In Adana, the maximum biomass yield was recorded before the first sampling date of this investigation (Nunn et al., under review), after which the yield declined steadily because drought in July and August ended the growth season. Interestingly, the standard genotype Mxg (OPM 9) performed best in terms of energy yield under the water-limited conditions in 2014 in Adana. The low irrigation levels applied to ensure survival of the crop will have influenced the performance of the genotypes. Indeed, Mxg is well known for sensitivity to drought (Clifton-Brown et al., 2002). However, from these observations, we conclude that while none of the genotypes tested here are optimally adapted to the climatic conditions of the Mediterranean area, *M. sinensis* coped better than the others.

In Moscow, the yield was comparatively low due to the short growing season determined by the more extreme continental climate (Figure 1B). This clearly shows that cold-tolerant genotypes, which start growing at lower temperatures, are required for such locations in order to make best use of the available vegetation period. However, Fonteyne et al. (2016) found that, for a C4 plant, miscanthus shows a comparatively high chilling tolerance. In Stuttgart, the mild continental climate with high water availability (Figure 1C) supported active growth for a longer period, resulting in higher autumn yields than in Moscow and Adana. Considerable genotypic differences were observed in Stuttgart, where the novel genotype OPM 6 performed best. This was mainly influenced by its high shoot density (Kalinina et al., under review). The effect of plant morphology on biomass yield demonstrates the opportunities of breeding high-yielding hybrids.

Earlier studies have found that moisture content is not only influenced by harvest date, but also determined by complex interactions between genotype and growth location environment (Iqbal and Lewandowski, 2014). Obviously, moisture content impacts the energy yield of combustion, since it directly reduces the heating value. However, the moisture content at final harvest is not only crucial for combustion quality, but also for safe storage of the biomass.

Genotypes with active senescence could help maintain sufficiently low moisture content at final harvest (Nunn et al., under review). This is especially relevant for locations with mild winters, as frost kills the aboveground biomass, thus accelerating senescence, initiating ripening, and drying the biomass (Robson et al., 2012). The largest genotypic differences in moisture content at final harvest were recorded in Adana, where almost no frost occurred over winter. At the other locations, only small differences in moisture content between genotypes were recorded, because there were sufficiently harsh frosts (below −3°C daily mean temperature). In Adana, only OPM 6, a *M. sinensis* x *M. sacchariflorus* hybrid, showed a sufficiently low moisture content of below 20% FM, while OPM 3, a pure *M. sacchariflorus* genotype, showed a particularly high moisture content. Genotypes with active senescence could also be useful at the Stuttgart location, because sufficient frosts to dry the crop below a moisture content of 20% do not occur every year. Iqbal and Lewandowski (2014) reported high differences in moisture content between single years at this location. Here, OPM 11 and 14 showed favorable development of moisture content until January, but after the February frost period, all genotypes had the same low moisture content at final harvest in March. In Adana, OPM 6 showed a gradual reduction in moisture content from autumn to spring. In Stuttgart, a similar decrease in moisture content from August until November was observed, but lodging hindered further drying. Genotypes with active senescence not only offer the potential to ensure sufficient drying even at locations with mild winters, but additionally allow optimization of harvest time for combustion (Iqbal et al., under review).

Moisture contents of above 60% have a greater impact on energy yield (Equation 3). Such high moisture contents were only recorded in August at Moscow and in August and September at Stuttgart. Drying over winter positively influenced the energy yield of combustion, but the improved biomass quality did not compensate for the yield losses e.g., due to leaf fall. This “trade-off” between biomass yield and quality is well known (Lewandowski et al., 2003; Cadoux et al., 2012) but has rarely been quantified due to the lack of serial harvests through the winter months. This paper quantifies the energy yield losses of delayed harvest in late winter compared to harvest at peak yield for the first time. Average energy yield losses were found to be 43% in Adana, 20% in Stuttgart and only 11% in Moscow. Some genotypes showed high energy yield losses over winter, such as OPM 3 in Adana (56%) and Moscow (53%), and OPM 11 in Stuttgart (36%). Genotype OPM 9 showed comparatively low losses at all locations (37% in Adana, 6% in Stuttgart and 4% in Moscow). However, as mentioned earlier, the biomass yield measurement of OPM 9 in Stuttgart was subject to technical variation, which could have negatively influenced these results from August to January. Other genotypes also showed contrasting results at the three locations, e.g., OPM 11 had high losses in Stuttgart (36%), but low losses in Moscow (4%) and Adana (36%). The yield losses could be associated with the leaf shares and OPM 9 showed the lowest leaf-to-stem ratio (Iqbal et al., under review). From an energy point of view, an earlier harvest would be theoretically advantageous for combustion, but is in conflict with biomass quality (see also Iqbal et al., under review).

The energy yield of anaerobic digestion is influenced more by DM yield than SMY, because SMY variations in the serial harvests were lower than initially expected. Similar findings have recently also been reported from other experiments (Wahid et al., 2015; Kiesel and Lewandowski, 2017). The biomass analyzed in the present study was milled (1 mm), which can affect the SMY. Frydendal-Nielsen et al. (2016) used a larger particle size than in our study and measured a lower SMY for miscanthus. In their study, pre-treatment increased the SMY of miscanthus significantly due to size reduction of the biomass particles. The SMY values in our paper show more the technical potential than the biogas yield, which would be obtained in full-scale biogas plants using chopped biomass. The current standard chip format for anaerobic digestion was developed for maize. Thus, presumably a pre-treatment would be required for miscanthus to achieve a similar SMY in full-scale biogas plants to that measured in our study. Various pre-treatment methods, including physical (e.g., milling, ultrasonic, steam-explosion), chemical (acid or alkaline), and biological methods (white and brown rot fungi, enzymes), to improve digestibility and methane yield of difficult and lignocellulosic substrates in anaerobic digestion are described in literature (Patinvoh et al., 2017). In recent years, suitable pre-treatment technology has become more available and is increasingly utilized in practice.

At the Adana and Stuttgart locations, the SMY decreased significantly with later harvest dates as the lignin content increased. Under anaerobic conditions, lignin is generally not digested and also inhibits the digestibility of other compounds (den Camp et al., 1988). Of all genotypes, OPM 9 had significantly lower SMY's, which correlates with the highest lignin content across all locations. Again, it is worth mentioning that the biomass was milled (1 mm) prior to the biogas batch test. This milling can be considered pre-treatment, which is known to increase digestibility of lignocellulosic biomass (Menardo et al., 2013; Frydendal-Nielsen et al., 2016). The SMY could have been positively affected by milling, especially for later harvest dates and genotypes with a higher degree of lignification. The effect of location on SMY is not clear. In the present study, Adana often had a significantly lower SMY, but also the lowest lignin content. Generally, drought conditions are expected to increase the lignin content (Le Gall et al., 2015). However, van der Weijde et al. (2017a) reported that drought conditions decreased lignin contents of miscanthus and increased the proportion of cellulose converted to ethanol. In our study, the drought conditions in Adana seemed to decrease the lignin content, but no positive effect on the SMY was observed.

Since biomass yield is more relevant than SMY for the energy yield of anaerobic digestion, the priority should be placed on harvesting at biomass peak yield. However, sufficient green-cutting tolerance is a prerequisite for this (Kiesel and Lewandowski, 2017). Green-cutting tolerance is assumed to be determined by relocation of carbohydrates from the aboveground biomass to the rhizome in late summer and early autumn (Purdy et al., 2015). By contrast, an increased nitrogen fertilizer application had almost no impact on the regrowth the following year of a 5-year-old Mxg crop in Stuttgart (Kiesel and Lewandowski, 2017). Green cuts also result in larger nutrient offtakes (Kiesel and Lewandowski, 2017), which need to be replaced, e.g., by digestate, to maintain long-term productivity of the crop.

Based on recent cutting trials with Mxg, a harvest in late October does not affect biomass yield the following year in Stuttgart, but earlier harvest can reduce DM yields by 40–60% (Kiesel and Lewandowski, 2017). Due to the harsh frost just before the sampling date in September in Moscow, it can be assumed that green harvest in late September or early October is feasible. In Adana, the season end was not defined by frost, but by drought in July and August. For this reason, it is questionable which harvest date would be tolerated by the crop here. Due to the favorable growing conditions before the drought period, the plants flowered very early, which may have induced senescence and carbohydrate relocation (Jensen et al., 2016). However, Purdy et al. (2015) observed no influence of flowering on carbohydrate relocation, but the growing conditions at their locations in UK were completely different from Adana. The steady biomass yield decrease in Adana shows there was no biomass growth after the drought period. This can be seen as an indication that an August green harvest could be tolerated by the crop here. Should this be the case, biomass yield losses and the necessary irrigation for crop survival during the drought period could be avoided. Cutting tolerance presumably also depends on genotype and location but this needs to be assessed for further genotypes and locations. A more detailed assessment of possible harvest dates in autumn (from September to late October) would be required to identify the feasibility of a harvest at biomass peak yield. For this reason, multi-location cutting tolerance studies should be performed for new leading genotypes such as OPM-6.

### Combustion vs. anaerobic digestion

Combustion has many advantages over anaerobic digestion. In this paper, the energy yield of anaerobic digestion, averaged over all harvest dates, was 35% lower than that of combustion. In addition, dry-harvested biomass can be stored easily for combustion, if the moisture is below 20%. Green-harvest could still be problematic for combustion due to content of critical elements and low ash melting temperature (Iqbal et al., under review). The identification of optimum harvest date requires a number of factors to be considered, including combustion technology applied, biomass yield, moisture content and various biomass quality aspects (Iqbal et al., under review). Therefore, it may not always be possible to harvest miscanthus at biomass peak yield for combustion and the state-of-the-art for most combustion applications is to delay harvest until March to improve biomass quality and moisture content. For this reason, it is perhaps less useful to compare energy yields for anaerobic digestion and combustion on the same harvest dates. If it is assumed that the crop tolerates green harvest in late August in Adana, anaerobic digestion delivers, on average, a 14% higher energy yield than combustion at final harvest in January. Harvest in late September for anaerobic digestion in Moscow and Stuttgart supplies only a 19 and 7% lower energy yield, respectively, than harvest for combustion in March. Even with delaying the harvest in Adana (September) and Stuttgart (October) to improve the cutting tolerance, the energy yield of anaerobic digestion is, on average, only 18% lower than that of combustion at final harvest.

### Recommendations for site-specific genotype choice

For both utilization options, genotypes with a high dry matter yield are required. Whereas, for anaerobic digestion the autumn biomass yield (often equal to peak yield) is crucial, for combustion a high biomass yield in late winter or spring is necessary. For this reason, genotypes such as OPM 9 with lower losses over winter (e.g., due to lower leaf share) are better suited for combustion. However, senescence of OPM 9 can be insufficient when winters are too mild, which leads to higher moisture content of the biomass accompanied by difficulties for harvest, storage and combustion. At such locations, high-yielding *M. sinensis* (e.g., OPM 11) or *M. sinensis* x *M. sacchariflorus* hybrids (such as OPM 6) could help ensure low moisture content at spring harvest. Since lodging occurred in OPM 6, this genotype cannot be recommended for combustion, because lodging makes the harvest more difficult and hinders drying of the biomass over winter. For anaerobic digestion, the impact of lodging is less critical, but still renders the harvest more difficult. Although OPM 6 lodged in Stuttgart, its utilization for anaerobic digestion still seems promising, because this genotype had a combination of high yield potential in autumn, high SMY and low lignin content. In Adana, OPM 11 appears promising due to its high yield in late summer and high SMY, but the cutting tolerance remains to be assessed. In Moscow, the *M. sacchariflorus* genotype OPM 3 performed best for anaerobic digestion, but cannot be recommended due to its creeping rhizome. For this reason, the second best-performing genotype OPM 6 is recommended for anaerobic digestion at this location.

Anaerobic digestion is a promising utilization option for miscanthus biomass, as the energy losses from conversion into gaseous fuel can be largely compensated for by avoiding biomass losses over winter. A short summary of the main findings is shown in Box 1. The storage of green miscanthus biomass via ensiling also appears feasible and can be further improved through the use of additives (Whittaker et al., 2016). To optimize the harvest date for anaerobic digestion, the cutting tolerance should be assessed at several locations and for multiple genotypes. Further, biogas plant technology needs to be adapted to process lignocellulosic miscanthus biomass or extended by suitable pre-treatment facilities. Encouraging practical experience has been gained using a MeWa Bio-QZ (ANDRITZ MeWa GmbH,Gechingen) at the full-scale research biogas plant of the University of Hohenheim. Anaerobic digestion of miscanthus has the potential to produce biogas more cheaply than other feedstocks and offers the co-benefit of easier nutrient recycling via digestate than via ash from combustion.

Box 1 **Short summary of the main outcomes:**

- Anaerobic digestion is a promising novel utilization pathway for miscanthus biomass, which provides both a higher value product and a high productivity per hectare
- Higher biomass yields due to harvest in autumn/at peak yield compensates largely for the conversion losses of anaerobic digestion. However, cutting tolerance of such novel genotypes needs to be assessed for a broad spectrum of locations.
- Biomass and energy losses due to delayed harvest for combustion, are the costs of quality improvements to meet the quality and storage requirements. Pre-winter harvest could increase energy yield of combustion, because higher moisture content is overcompensated by higher biomass yields. However, adapted and suitable technology for storage and combustion of wet biomass are required.
- Environmental impacts (soil organic carbon, biodiversity) of pre-winter harvest needs to be assessed, since mulch layer is likely to decrease due to reduced leaf fall and reduced winter-cover.
- Combustion and anaerobic digestion both require genotypes with a high biomass production. However, for combustion low yield losses over winter and a high stability of the crop (no lodging) are of importance, while for anaerobic digestion cutting tolerance and easier digestibility (low lignin content) are important.

## Author contributions

AK and IL were leading the preparation and writing of this paper. JC, LT, and all other co-authors contributed to the writing of the manuscript and in discussing the results. CN collected and provided data of the multilocation trials relevant for the preparation of this paper. YI, MÖ, IT, and OK supported and conducted sampling of the field trials.

### Conflict of interest statement

The authors declare that the research was conducted in the absence of any commercial or financial relationships that could be construed as a potential conflict of interest.
