# Supplementary material for: Site-Specific Management of Miscanthus Genotypes for Combustion and Anaerobic Digestion: A Comparison of Energy Yields
Source: Front Plant Sci. 2017 Mar 17;8:347. doi: 10.3389/fpls.2017.00347 (PMC5355453; doi:10.3389/fpls.2017.00347)
Supplement: Supplementary file 1 [file Table1.DOCX]

**Supplement 1** Means for each analysed trait of each genotype at each location over all sampling dates at the respective location. Means of the same trait and at the same location with the same italic, lower or upper case letter in brackets differ not significantly at a level of probability of α = 0.05.

| **Genotype** | | **Location** | | **Yield**  **[t DM ha^-1^]** | | **Dry matter content**  **[% FM]** | | **Ash content**  **[% DM]** | | **Cellulose content**  **[% DM]** | | **Hemicellulose content**  **[% DM]** | | **Lignin content**  **[% DM]** | | **SMY**  **[ml (g oDM)^-1^]** | | **Methane yield per hectare**  **[m^3^ ha^-1^]** | | **Net energy yield anaerobic digestion**  **[GJ ha^-1^]** | | **Net energy yield combustion**  **[GJ ha^-1^]** | |
| --- | --- | --- | --- | --- | --- | --- | --- | --- | --- | --- | --- | --- | --- | --- | --- | --- | --- | --- | --- | --- | --- | --- | --- |
| Adana | OPM 3 | | 7.6 (b) | | 54.9 (b) | | 6.0 (c) | | 36.1 (d) | | 28.1 (b) | | 6.5 (c) | | 277.2 (a) | | 1977 (b) | | 70.9 (b) | | 112.9 (b) | |  |
|  | OPM 6 | | 12.8 (a) | | 68.8 (a) | | 6.5 (bc) | | 39.3 (bc) | | 29.2 (a) | | 7.1 (b) | | 268.6 (b) | | 3215 (ab) | | 115.4 (ab) | | 200.0 (a) | |  |
|  | OPM 9 | | 18.3 (a) | | 59.4 (b) | | 4.5 (d) | | 41.2 (a) | | 26.2 (c) | | 9.2 (a) | | 251.7 (c) | | 4391 (a) | | 157.5 (a) | | 281.7 (a) | |  |
|  | OPM 11 | | 15.9 (a) | | 57.4 (b) | | 8.5 (a) | | 40.2 (ab) | | 29.4 (a) | | 6.7 (bc) | | 277.5 (a) | | 4017 (a) | | 144.2 (a) | | 230.1 (a) | |  |
|  | OPM 14 | | 12.7 (a) | | 57.0 (b) | | 7.5 (ab) | | 38.4 (c) | | 29.6 (a) | | 6.8 (bc) | | 281.7 (a) | | 3289 (ab) | | 118.0 (ab) | | 185.5 (ab) | |  |
| Moscow | OPM 3 | | 6.1 (AB) | | 52.8 (A) | | 3.2 (A) | | 39.8 (B) | | 30.0 (C) | | 7.9 (B) | | 283.5 (A) | | 1683 (AB) | | 60.4 (AB) | | 82.4 (AB) | |  |
|  | OPM 6 | | 7.9 (A) | | 56.5 (A) | | 3.6 (A) | | 39.0 (BC) | | 33.3 (B) | | 7.2 (C) | | 283.8 (A) | | 2165 (A) | | 77.7 (A) | | 115.5 (A) | |  |
|  | OPM 9 | | 6.9 (AB) | | 52.5 (A) | | 3.4 (A) | | 42.2 (A) | | 27.7 (D) | | 10.5 (A) | | 255.0 (B) | | 1698 (AB) | | 60.9 (AB) | | 97.6 (AB) | |  |
|  | OPM 11 | | 5.0 (B) | | 56.2 (A) | | 3.5 (A) | | 39.6 (B) | | 33.9 (AB) | | 7.8 (B) | | 277.4 (A) | | 1347 (B) | | 48.3 (B) | | 73.5 (B) | |  |
|  | OPM 14 | | 5.4 (AB) | | 55.2 (A) | | 3.6 (A) | | 38.1 (C) | | 34.5 (A) | | 7.2 (C) | | 282.6 (A) | | 1472 (B) | | 52.8 (B) | | 79.2 (B) | |  |
| Stuttgart | OPM 3 | | 17.6 (*a*) | | 49.6 (*c*) | | 3.8 (*bc*) | | 44.7 (*a*) | | 26.2 (*c*) | | 8.9 (*a*) | | 286.0 (*a*) | | 4831 (*ab*) | | 173.4 (*ab*) | | 248.6 (*ab*) | |  |
|  | OPM 6 | | 20.8 (*a*) | | 58.0 (*a*) | | 3.5 (*c*) | | 42.8 (*bc*) | | 30.0 (*b*) | | 7.8 (*b*) | | 286.4 (*a*) | | 5744 (*a*) | | 206.1 (*a*) | | 319.7 (*a*) | |  |
|  | OPM 9 | | 15.0 (*ab*) | | 51.8 (*b*) | | 4.0 (*ab*) | | 43.8 (*ab*) | | 26.9 (*c*) | | 9.5 (*a*) | | 275.6 (*b*) | | 3959 (*bc*) | | 142.1 (*bc*) | | 217.5 (*bc*) | |  |
|  | OPM 11 | | 16.5 (*ab*) | | 56.6 (*a*) | | 3.9 (*bc*) | | 42.3 (*c*) | | 31.3 (*a*) | | 7.7 (*b*) | | 287.7 (*a*) | | 4549 (*ab*) | | 163.2 (*ab*) | | 245.5 (*ab*) | |  |
|  | OPM 14 | | 11.4 (*b*) | | 57.7 (*a*) | | 4.4 (*a*) | | 40.6 (*d*) | | 31.8 (*a*) | | 7.3 (*b*) | | 286.7 (*a*) | | 3126 (*c*) | | 112.2 (*c*) | | 170.8 (*c*) | |  |
